# Mediating role of obesity on the association between disadvantaged neighborhoods and intracortical myelination

**DOI:** 10.21203/rs.3.rs-2592087/v1

**Published:** 2023-03-16

**Authors:** Lisa Kilpatrick, Keying Zhang, Tien Dong, Gilbert Gee, Hiram Beltran-Sanchez, May Wang, Jennifer Labus, Bruce Naliboff, Emeran Mayer, Arpana Gupta

**Affiliations:** University of California Los Angeles

## Abstract

We investigated the relationship between neighborhood disadvantage (area deprivation index [ADI]) and intracortical myelination (T1-weighted/T2-weighted ratio at deep to superficial cortical levels), and the potential mediating role of the body mass index (BMI) and perceived stress in 92 adults. Worse ADI was correlated with increased BMI and perceived stress (p's<.05). Non-rotated partial least squares analysis revealed associations between worse ADI and decreased myelination in middle/deep cortex in supramarginal, temporal, and primary motor regions and increased myelination in superficial cortex in medial prefrontal and cingulate regions (p<.001); thus, neighborhood disadvantage may influence the flexibility of information processing involved in reward, emotion regulation, and cognition. Structural equation modelling revealed increased BMI as partially mediating the relationship between worse ADI and observed myelination increases (p=.02). Further, trans-fatty acid intake was correlated with observed myelination increases (p=.03), suggesting the importance of dietary quality. These data further suggest ramifications of neighborhood disadvantage on brain health.

## Introduction

Living in a disadvantaged neighborhood (area deprivation) is linked to worse health outcomes, including poor brain health^1^. Disadvantaged neighborhoods can be stressful, and which can then alter brain structure and function, including decreased brain volume^2^. The key mechanisms that underlying the link between neighborhood conditions and brain health remain unclear, but one possible pathway might be through obesity. People who live in disadvantaged neighborhoods are at higher risk of obesity due to the poor quality of available foods and environments that hamper physical activity^3, 4, 5^. In particular, neighborhood disadvantage is associated with an increased intake of calories from trans-fatty acids (TFAs) and sodium^6^. TFAs (high in fried fast food) are known to contribute to obesity, especially abdominal obesity^7, 8^. Additionally, chronic neighborhood stressors (allostatic load) can impact eating behaviors^9, 10^, increasing desire for highly palatable, but unhealthy foods used as a coping response^11, 12^. Numerous neuroimaging studies have demonstrated that stress can alter brain structure and function, leading to food cravings, contributing to an increased risk for obesity^13, 14, 15^. Further, high body mass index (BMI) has been shown to mediate the impact of living in a disadvantaged neighborhood on reduced brain volume, suggesting its importance in the negative impact of neighborhood disadvantage on brain health^16^.

Neighborhood disadvantage, high BMI, and chronic stress have also been shown to impact intracortical myelination^17, 18, 19^. Intracortical myelination, which refers to the myelination of axons in the cortical gray matter, affects the timing and integration of signals from multiple axons, and is critical for neural synchrony and fine-tuning of cortical circuits, affecting cognitive functioning^20, 21, 22^. Additionally, intracortical myelination is actively involved in brain remodeling and plasticity throughout the lifespan^23, 24^. Myelinated components vary by cortical layer, with a large fraction of intracortical myelin ensheathing the axons of inhibitory internerons in upper cortical layers (layers 1-3); specifically, parvalbumin-positive basket cells, which play an important role in gamma network oscillations that support a variety of cognitive processes^25^. In deeper cortical layers, myelin predominantly ensheaths non-gamma-aminobutyric acid (non-GABA) axons, presumably from long-distance excitatory pyramidal neurons, which integrate thousands of synaptic inputs and act on targets in other cortical regions and subcerebral structures^26, 27, 28^. Further, cortical layers vary in terms of specific inputs and outputs and information processing functions (e.g., subcortical vs. intercortical input, feedback vs. forward processes)^29^. Accordingly, examining intracortical myelination at different cortical layers can inform how alterations in cell populations, processes, and communication routes may be affected by adverse or stressful environments, such as living in a disadvantaged neighborhood.

We, therefore, investigated the relationship between the area deprivation index (ADI) and intracortical myelination at multiple cortical levels, as well as the role of potential mediators, including BMI and stress (**Graphical Abstract**). In addition, we investigated the relationship between TFA intake and intracortical myelination in a subset of participants with diet data. We hypothesized that worse ADI would be associated with higher BMI and stress levels, with negative effects on intracortical myelination in reward-related, emotion regulation, and cognitive regions, related to poor dietary quality characterized by high TFA intake.

## Results

### Sample characteristics

Participant characteristics, including TFA intake, are summarized in Table 1. In this sample, ADI was positively correlated with BMI (r=0.27, p=0.01) and the Perceived Stress Scale (PSS) score (r=0.22, p=0.04), but BMI and PSS were not correlated (r=0.04, p=0.73).

### Regions showing a relationship between ADI and intracortical myelination

**Figure 1:**
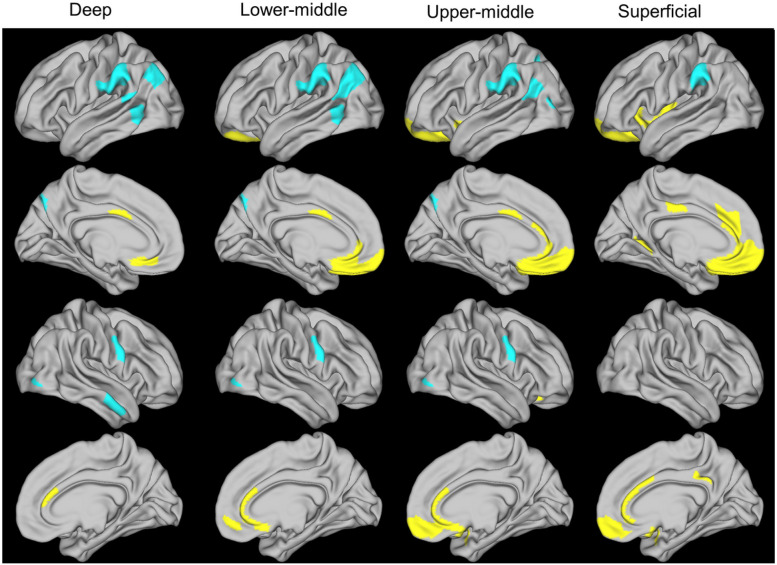
Partial least squares analysis of the relationship between worse ADI and intracortical myelination at 4 cortical ribbon levels The 4 cortical ribbon levels were as follows: deep, 5%-25% of cortical thickness from white-pial boundary; lower-middle, 30%-50%; upper-middle, 55%-75%, superficial, 80%-100%. Worse ADI was associated with increased myelination (yellow) in medial prefrontal and cingulate regions, and decreased myelination (blue) in supramarginal, middle temporal, and primary motor regions (p<.001). ADI, area deprivation index

Non-rotated partial least squares correlational (PLSC) analysis revealed worse ADI as associated with increased myelination in medial prefrontal and cingulate regions (hereupon referred to as ADI-positive regions), involved in reward-related processing, emotional regulation, and higher cognition^30, 31, 32^, and decreased myelination in supramarginal, middle temporal, and primary motor regions (hereupon referred to as ADI-negative regions), components of the extended mirror system involved in social interaction (p<0.001) (Figure 1)^33, 34, 35^. Regions with a positive association were more extensive at middle/superficial cortical levels, while those with a negative association were more extensive at middle/deep cortical levels. Intracortical myelination was averaged across all ADI-positive regions, and across all ADI-positive regions, for further analysis.

### Mediation of the relationship between ADI and intracortical myelination

**Figure 2:**
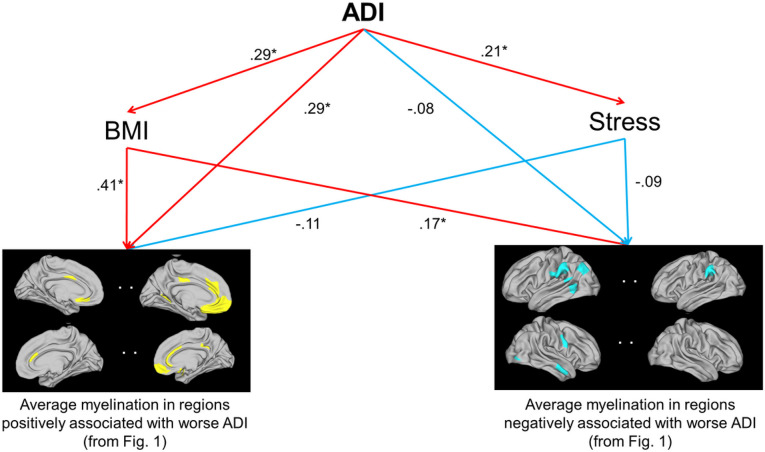
Structural equation modeling analysis of the mediation of the relationships between worse ADI and intracortical myelination. BMI partially mediated the relationship between ADI and intracortical myelination in regions positively associated with worse ADI. *p<.05; ADI, area deprivation index; BMI, body mass index

The final model in the structural equation modelling (SEM) analysis is shown in Figure 2; for simplicity, sex and age are not shown in the model, but were used as control variables. The model had a high chi-squared p-value (χ^2^(2)=0.025, p=0.988), comparative fit index of 1.0, and standardized root mean square residual of 0.002, indicating good model fit. BMI had significant positive direct effects on both brain variables (ADI-positive regions: p<0.001; ADI-positive regions: p=0.03). Additionally, BMI partially mediated the relationship between ADI and the average intracortical myelination in ADI-positive regions, but not ADI-negative regions (indirect effect of ADI on ADI-positive regions via BMI: p=0.02; on ADI-negative regions: p=0.09). Although stress (PSS score) had a negative direct effect on both brain variables, statistical significance was not reached (ADI-positive regions: p=0.33; ADI-positive regions: p=0.31). Stress also failed to reach significance as a mediator of the relationship between ADI and the average intracortical myelination in these regions (indirect effect of ADI on ADI-positive regions via stress: p=0.36; on ADI-negative regions: p=0.32).

### Relationship between TFA intake and intracortical myelination

Correlations between TFA intake and intracortical myelination are shown in Table 2. Significant positive correlations were observed between the average intracortical myelination in ADI-positive regions and trans-octadecenoic (elaidic) acid and total TFA intake (p’s<0.05). No significant correlations were observed for the average intracortical myelination in ADI-positive regions.

## Discussion

We examined the relationship between ADI and myelin content, as assessed by the T1-weighted/T2-weighted (T1w/T2w) ratio, at different levels of the cortical ribbon, as well as potential mediation by factors associated with ADI, namely, BMI and stress. We found that ADI was associated with increased myelination in medial prefrontal and cingulate cortices at more superficial levels (we refer to these regions as ADI-positive regions); these regions are involved in reward-related, emotion regulation, and higher cognitive processes^30, 31, 32^. This association was partially mediated by increased BMI. We also found ADI associated with decreased myelination in supramarginal, middle temporal, and primary motor cortices at deeper levels (we refer to these regions as ADI-negative regions); these regions are components of the mirror neuron system, involved in social interaction^33, 34, 35^. BMI did not appear to mediate this relationship. Further, perceived stress was associated with ADI but not myelination. Cortical layers differ in the type of axons myelinated and information processing functions, with superficial cortex receiving top-down information that can modulate feed-forward and feed-back processes in deeper layers^26, 27, 28, 29^. Thus, our findings provide new insights as to the nature of affected information processing pathways under worse ADI, as discussed below.

### ADI and increased superficial intracortical myelination

In the present study, worse ADI was associated with increased myelination signal in more superficial cortex in medial prefrontal and cingulate regions. These results are largely similar to a previous developmental study on the relationship between disadvantage due to low socioeconomic status (SES) and intracortical myelination as assessed by the T1w/T2w ratio. This previous study found that lower parental SES was associated with increased intracortical myelination in frontal, temporal, medial parietal, and occipital regions in children and adolescents^36^. Thus, disadvantage due to ADI or individual SES may be associated with increased intracortical myelination in overlapping regions.

The maturation of intracortical myelination is protracted in humans, especially in prefrontal regions, peaking at 30-45 years of age depending on the brain region^37^. Accordingly, intracortical myelination within a normative range may be needed for optimal function, with both reduced and excessive myelination being problematic^38^. Consistent with the latter, animal studies have found that under conditions of excessive myelination (i.e., beyond axonal demand) mistargeting to cell bodies occurs readily^39^. Thus, our finding that worse ADI and increased BMI are associated with increased myelination in more superficial cortex in medial prefrontal and cingulate regions may imply excessive and disorganized myelination in the upper layers of the cortex. Superficial cortex receives top-down information from subcortical and cortical regions and is thought to enable flexible and state-dependent processing of feedforward sensory input arriving in deeper layers^29, 40^. One may speculate that myelination mistargeting in superficial cortex could negatively influence the context and flexibility of information processing in affected regions, which in the present study, comprised the prefrontal and cingulate cortices. Given the involvement of these regions in reward-related processing, emotional regulation, and higher cognition, this interpretation is similar to previous behavioral studies showing an impact of neighborhood disadvantage on these functions throughout the lifespan^41, 42, 43, 44^.

However, in contrast with the current results, studies using markers of intracortical myelination other than the T1w/T2w ratio have mainly found reductions associated with neighborhood or socioeconomic disadvantage. For example, a study using magnetization transfer as a myelin-sensitive marker, found that living in a disadvantaged neighborhood before the age of 12 years was associated with slower myelin growth in adolescents and young adults in sensorimotor, cingulate, and prefrontal cortices^18^. Another study using magnetization transfer found that SES in adulthood was associated with decreased entorhinal cortical myelination^45^. Although the T1w/T2w ratio is sensitive to intracortical myelin content, accumulating evidence suggests that it is not a straightforward proxy for intracortical myelin^46, 47^. Given our finding that increased intracortical myelination in more superficial, but not deeper, cortical levels of prefrontal and cingulate regions was mediated by BMI, we entertained an alternative explanation of our results. We considered it possible that the T1w/T2w signal at the upper BMI range was affected by a fatty acid-rich cortical environment, with lipid droplet accumulation and lipid-laden astrocytes, due to blood-brain barrier disruption and increased transport of fatty acids under obese conditions^48, 49, 50^. In support of this, we found that total TFA intake, largely driven by trans-octadecenoic (elaidic) acid intake, was correlated with increased superficial cortical myelination in ADI-positive regions. Although industrial TFAs such as partially hydrogenated oil have been banned in the United States because of health concerns (effective 2020), the process of repeatedly cooking oil at high temperatures can cause high levels of TFAs in fried fast foods^51^. Additionally, some meat and dairy products naturally contain small amounts of TFA^52^. Higher intake of TFA, including elaidic acid, is associated with an increased risk of dementia^53, 54^. TFAs can be incorporated into cell membranes, including myelin^55^. Poor-quality diet, such as a diet high in fried fast foods, is thought to be one of the factors of worse ADI that contributes to obesity and worse health outcomes^3, 4, 5, 56^. Thus, our results could suggest that a diet high in TFA under worse ADI may create a fatty acid-rich environment in superficial cortical layers, become incorporated into cell membranes and disrupt information processing in affected regions.

### ADi and decreased intracortical myelination

We also found that worse ADI was associated with decreased myelination in middle/deep cortex in supramarginal, middle temporal, and primary motor regions. These regions are components of the mirror neuron system, involved in understanding the actions of others and in interpersonal coordination in activities (e.g. imitation, cooperation)^33, 34, 35^. As middle/deep levels were involved, feed-forward and feed-back processes and intercortical and subcortical-cortical communication in these regions may be affected with worse ADI^29^. Animal studies suggest that myelin plasticity is a major component in the response to stress^57^. In addition, a previous study using the T1w/T2w ratio as a measure of intracortical myelination found that higher perceived stress was associated with lower intracortical myelination in the right supramarginal gyrus^19^. However, in the present study, perceived stress was not significantly associated with decreased myelination in ADI-negative regions, which included the left supramarginal gyrus. Thus, mediation of the association between worse ADI and decreased intracortical myelination in these regions remains unclear.

### Limitations

The present study has several limitations. Although the T1w/T2w ratio is sensitive to myelin, it may not be a straightforward proxy, as it does not correlate well with myelin-related gene expression and other measures^58^. Confirmation studies using other acquisition protocols sensitive to intracortical myelin are needed. Additionally, we assessed current ADI at one point in time; we did not have information regarding the length of residence, nor did we have historical data on ADI in younger ages. A previous study found that, although both child and adult SES showed correlations with intracortical myelination, childhood SES showed robust associations even after controlling for adult SES, suggesting a lasting imprint, which may also hold for neighborhood-level factors such as ADI^42, 45^.

## Conclusions

We found that worse ADI was associated with decreased myelination in middle/deep cortex in supramarginal, middle temporal, and primary motor regions, potentially impacting intercortical and subcortical-cortical communication of the mirror neuron system, important for understanding the actions of others and cooperative behavior. ADI was also associated with increased myelination in the superficial cortex in medial prefrontal and cingulate regions, which was partially mediated by increased BMI. Further, this increased myelination was positively correlated with TFA intake. Thus, obesogenic features of neighborhood disadvantage may disrupt the flexibility of information processing involved in reward, emotion regulation, and cognition. These results provide new information regarding the ramifications of living in a disadvantaged neighborhood on brain health. Further research on the mediating factors involved in the impact of ADI on the brain during development and adulthood is needed.

## Methods

### Participants

Participants comprised 92 adults (27 men; 65 women) recruited from the Los Angeles area who completed a neuroimaging session including both T1-weighted (T1w) and T2-weighted (T2w) scans (enabling the calculation of intracortical myelination) and provided residential address information. Exclusion criteria were as follows: major neurological condition, current or past psychiatric illness, vascular disease, weight loss/abdominal surgery, substance use disorder, use of medications that interfere with the central nervous system, pregnant or breastfeeding, strenuous exercise regimen (> 8 h/week of continuous exercise), weight > 400 pounds, or metal implants. In addition, individuals with poor quality images were excluded. Image quality was evaluated using Qi1, which reflects the proportion of voxels with intensity corrupted by artifacts normalized by the number of voxels in the background, from the MRI Quality Control tool (MRIQC)^30^. Qi1 > 0.00506 for T1w images and > 0.0030 for T2w images was considered to indicate poor image quality^31^.

All procedures were approved by the Institutional Review Board at the University of California, Los Angeles’s Office of Protection for Research Subjects (Nos. 16–000187, 15-001591). All participants provided written informed consent.

### Assessments

Basic demographic data, as well as weight and height, were collected. BMI was calculated as weight divided by the square of the height (kg/m^2^). ADI was originally developed by the Health Resources and Services Administration several decades ago and is updated periodically. We used the 2020 ADI in the Neighborhood Atlas^®^^32^, based on the residential address provided by the participant. This atlas ranks census block groups, which are considered as similar to neighborhoods, on 17 neighborhood-level measures reflecting income, education, employment, and housing quality, within state and nationally. We employed the California State ADI, which is provided in deciles (scores range 1–10), with higher values indicating greater deprivation/disadvantage.

Participants also completed the Perceived Stress Scale (PSS), which is a 10-item questionnaire that assesses feelings of stress during the prior month^33^. Scores range from 0 to 40, with higher scores indicating greater stress. Diet information was collected using the VioScreen Graphical Food Frequency System (Viocare Technologies, Inc., Princeton, NJ), and was available in a subset of participants (N = 81) as 11 participants did not provide this information. The VioScreen System provides information on nutrient intake, including fatty acid intake. We focused on the intake of individual TFAs (trans-hexadecenoic acid, trans-octadecenoic acid [elaidic acid], and trans-octadecadienoic acid [linolelaidic acid]), as well as the total TFA intake. TFA intake was measured as a component of a poor-quality diet known to contribute to obesity, especially abdominal obesity, and have harmful effects on brain cell membranes, including myelin^7, 8, 34^.

### Imaging acquisition and preprocessing

T1w and T2w structural images were obtained for the non-invasive assessment of intracortical myelination using a 3.0T Siemens Prisma MRI scanner (Siemens, Erlangen, Germany). Spin echo fieldmaps were also acquired in anterior-posterior and posterior-anterior directions for distortion correction. The acquisition parameters for high-resolution T1w images were as follows: echo time, 1.81 ms; repetition time, 2500 ms; slice thickness, 0.8 mm; number of slices, 208; voxel matrix, 320×300; and voxel size, 1.0×1.0×0.8 mm. The parameters for the T2w images were as follows: echo time, 564 ms; repetition time, 3200 ms; slice thickness, .8 mm; number of slices, 208; voxel matrix, 320×300; and voxel size, 1.0×1.0×0.8 mm.

Imaging data were preprocessed using Human Connectome Project (HCP) pipelines, including volume segmentation and cortical surface reconstruction with FreeSurfer 6.0^35, 36^. Intracortical myelination was estimated by the T1w/T2w ratio at multiple levels within the cortical ribbon^37, 38^. Specifically, the T1w/T2w ratio was calculated at 5% increments in cortical thickness from the gray-white boundary to the gray-cerebrospinal fluid boundary and averaged within 4 cortical ribbon levels as follows: 5%-25% (deep cortex), 30%-50% (lower-middle cortex), 55%-75% (upper-middle cortex), and 80%-100% (superficial cortex). The 4 resulting myelin maps were parcellated using the HCP multi-modal parcellation 1.0 atlas, resulting in 360 cortical regions with 4 intracortical myelination estimates each^39^.

### Statistical analysis

Partial correlation coefficients, controlling for sex and age, were calculated to determine individual factors associated with worse ADI using SPSS version 28 (IBM Crop., Albany, NY, USA), with bootstrapping (5000 samples). P-values < .05 were considered statistically significant.

Non-rotated partial least squares correlational (PLSC) analysis was applied to identify regions correlated with ADI according to cortical level, using freely available code (http://www.rotman-baycrest.on.ca/pls)^40^. PLSC is a multivariate analytical technique that identifies weighted patterns of variables in two blocks of variables that maximally covary with each other^40, 41^. In this study, one block comprised demographic variables (ADI, age, sex) and one block comprised parcellated intracortical myelin values (at a specific cortical level). Weights were preset to identify brain regions sensitive to ADI but not sex or age. Reliability of identified regions was assessed using bootstrap estimation (5000 samples); regions with a bootstrap ratio > 3.1 (p < .001) were considered significant. Myelin data was extracted from significant brain clusters to calculate an average of all regions/levels with a positive relationship with ADI and an average of all regions/levels with a negative relationship with ADI, for further analysis.

Structural equation modeling (SEM) was applied to investigate the mediation of relationships between ADI and significant findings from the PLSC analysis. SEM was performed in R Studio using the lavaan package^42^. Input variables comprised ADI, BMI, PSS score, average myelination in ADI-positive regions, and average myelination in ADI-negative regions, as well as sex and age as control variables. Data were standardized prior to fitting the model. Missing values were estimated using the maximum likelihood (4 participants had missing PSS scores). Model fit was assessed using the chi-squared p-value, comparative fit index, and standardized root mean square residual. Model paths with a p-value < .05 were considered significant.

## Figures and Tables

**Table 1.** Participant characteristics

Variable	N (%) or Mean±SD
**Male**	27 (29.3%)
**Age (yrs)**	28.0±10.3
**Hispanic (%)**	37 (40.2%)
**Race (%)**	
Asian	34 (37.0%)
Black	5 (5.4%)
White	28 (30.4%)
Mixed	11 (112.0%)
Other	19 (20.7%)
**Income (%)**	
<20k	8 (8.7%)
20k-39k	14 (15.2%)
40k-59k	15 (16.3%)
60k-79k	17 (18.5%)
80k +	32 (34.8%)
**Body mass index (kg/m^2^)**	24.9±4.7
**Perceived Stress Scale score**	15.05±7.5
**National ADI**	11.4±10.6
**California ADI**	4.0±2.4
**Trans-fatty acid intake (unit?)**	
trans-hexadecenoic acid	.03±.02
trans-octadecenoic (elaidic) acid	1.38±.80
trans-octadecadienoic (linolelaidic) acid	.24±.14
Total	1.65±.93

Data are mean±standard deviation, unless otherwise indicated.

ADI, area deprivation index

**Table 2.** Correlations between TFA Intake and intracortical myelination, controlling for sex and age

	trans- hexadecenoic acid	trans-octadecenoic (elaidic) acid	trans-octadecadienoic (linolelaidic) acid	Total TFA
Regions positively associated with ADI	.10	**.29** *	.18	**.28** *
Regions negatively associated with ADI	.09	.20	.12	.19
BMI	.24*	.27*	.24*	.27*

ADI, area deprivation index; BMI, body mass index; TFA, tans-fatty acid

* p<.05

## Data Availability

The datasets generated during and/or analyzed during the current study are not publicly available due to an ongoing collaboration with multiple principal investigators involving participant identifiers at the G. Oppenheimer Center for Neurobiology of Stress and Resilience. However, data is available from the corresponding author on reasonable request.
